# Assessment of mitochondrial function and its prognostic role in sepsis: a literature review

**DOI:** 10.1186/s40635-024-00694-9

**Published:** 2024-11-25

**Authors:** Wagner Nedel, Nathan Ryzewski Strogulski, Afonso Kopczynski, Luis Valmor Portela

**Affiliations:** 1 Intensive Care Unit, Grupo Hospitalar Conceição (GHC), Porto Alegre, Brazil; 2 Laboratory of Neurotrauma and Biomarkers, Departamento de Bioquímica, Instituto de Ciências Básicas da Saúde, Universidade Federal do Rio Grande do Sul (UFRGS), Porto Alegre, Brazil; 3 School of Biochemistry and Immunology, Trinity Biomedical Sciences Institute, Trinity College Dublin, University of Dublin, Dublin, Ireland; 4 Programa de Pós-Graduação Em Bioquímica, Universidade Federal do Rio Grande do Sul (UFRGS), Porto Alegre, Brazil; 5 Unidade de Terapia Intensiva, Hospital Nossa Senhora da Conceição, Av Francisco Trein, 596-primeiro andar, Porto Alegre, RS Brazil

**Keywords:** Sepsis, Mitochondria, Mitochondrial dysfunction, Oxidative phosphorylation, Inflammation, Respirometry

## Abstract

Sepsis is characterized by a dysregulated and excessive systemic inflammatory response to infection, associated with vascular and metabolic abnormalities that ultimately lead to organ dysfunction. In immune cells, both non-oxidative and oxidative metabolic rates are closely linked to inflammatory responses. Mitochondria play a central role in supporting these cellular processes by utilizing metabolic substrates and synthesizing ATP through oxygen consumption. To meet fluctuating cellular demands, mitochondria must exhibit adaptive plasticity underlying bioenergetic capacity, biogenesis, fusion, and fission. Given their role as a hub for various cellular functions, mitochondrial alterations induced by sepsis may hold significant pathophysiological implications and impact on clinical outcomes. In patients, mitochondrial DNA concentration, protein expression levels, and bioenergetic profiles can be accessed via tissue biopsies or isolated peripheral blood cells. Clinically, monocytes and lymphocytes serve as promising matrices for evaluating mitochondrial function. These mononuclear cells are highly oxidative, mitochondria-rich, routinely monitored in blood, easy to collect and process, and show a clinical association with immune status. Hence, mitochondrial assessments in immune cells could serve as biomarkers for clinical recovery, immunometabolic status, and responsiveness to oxygen and vasopressor therapies in sepsis. These characteristics underscore mitochondrial parameters in both tissues and immune cells as practical tools for exploring underlying mechanisms and monitoring septic patients in intensive care settings. In this article, we examine pathophysiological aspects, key methods for measuring mitochondrial function, and prominent studies in this field.

## Introduction

Sepsis is a major health problem worldwide associated with a dysregulated host response to an infection that results in life-threatening tissue damage and organ failure [1]. An inappropriate response to infection leads to cellular dysfunction and, ultimately, organ failure [2, 3]. There have been great efforts in the recognition and prompt treatment of sepsis, especially with regard to early antibiotic administration, hemodynamic resuscitation, and evacuation of infection foci [1, 4]. However, the clinical trajectory of sepsis and the underlying cause of mortality remain incompletely understood. While microcirculatory failure is observed in many septic patients, mortality often occurs despite adequate resuscitation, normal or even elevated cardiac output, and sufficient cellular oxygen levels, suggesting appropriated microcirculation [5]. Fatal sepsis is marked by an inability to resolve systemic inflammation, as cytokines released into circulation activate inflammatory cells in distant tissues [6]. This widespread inflammatory response leads to organ injury and dysfunction [7], likely due to impaired oxygen utilization by mitochondria. Within this framework, mitochondrial dysfunction has also emerged as a critical mechanistic factor linked to cellular death and is increasingly recognized as a contributor to organ failure in sepsis [2].

Mitochondria are highly specialized organelles that support energy dependent processes through adenosine triphosphate (ATP) biosynthesis coupled with metabolic substrates and oxygen utilization [8]. It is present in almost all eukaryotic organisms, and have their own genetic code, mitochondrial DNA (mtDNA) [5]. Given their role as a central hub for various cellular functions, mitochondrial alterations induced by sepsis may hold significant pathophysiological implications that impact clinical outcomes. Even minor changes in mitochondrial integrity can impact many aspects of cellular homeostasis [8]. Beyond generating ATP through oxidative phosphorylation (OXPHOS) of adenosine diphosphate (ADP), mitochondria also play key roles in the production and detoxification of reactive oxygen species (ROS), calcium homeostasis, apoptotic signaling, synthesis and breakdown of metabolites, and intracellular organelle transport [9]. Pathological processes can disrupt these diverse mitochondrial functions, leading to inhibited electron transport due to depletion of essential electron transport chain (ETC) proteins, proton leakage across the inner membrane, and impaired utilization of molecular oxygen. This disruption leads to exacerbated production of toxic reactive species and lower ATP biosynthesis [5]. Collectively, any alteration in these parameters can be defined as mitochondrial dysfunction. In sepsis, mitochondrial dysfunction has been investigated as a biomarker for identifying patients at risk of progressing to organ failure, and for monitoring responses to therapeutic management, including oxygen therapy, antibiotics, vasopressors, and volume resuscitation [6, 10, 11]. For example, studies have shown that the acute phase of sepsis is characterized by reduced mitochondrial biogenesis, increased ROS production, and impaired OXPHOS [7, 12–14]. Reduced mitochondrial metabolism has been correlated with poorer prognosis in sepsis and organ-specific injuries [15–20]. Notably, the complexity of the mitochondria's “fan in and fan out” interactions may further facilitate the discovery of new mechanistic targets for sepsis treatment and the measurement of mitochondrial metabolism through cellular respiration.

Actually, mitochondrial metabolism has been shown to be severely impaired during the early phase of an acute disease via several pathways. Therefore, mitochondrial alterations offers the opportunity to identify patients at risk of progressing in their organ failure, and may unravel mechanistic targets for future therapeutic strategies in sepsis management.

In this review, we explore three key points related to mitochondria and sepsis: (i) the interaction between mitochondrial metabolism and the immune response; (ii) the feasible methods for measuring “mitochondrial dysfunction”; and (iii) the clinical associations between mitochondrial dysfunction and clinical outcomes of critically ill patients.

## Interaction between mitochondrial metabolism and inflammatory activity in sepsis

The hallmark of the immune response in sepsis is an imbalance between the pro-inflammatory and the compensatory anti-inflammatory responses [21, 22]. This often leads to immunoparalysis among critically ill patients, rendering them more susceptible to additional infections associated with higher mortality rates [23]. The root cause of immune dysfunction remains a matter of debate [22], however, recent findings suggest that the metabolic pathways in immune cells mediates both pro- and anti-inflammatory responses [24], an area known as “immunometabolism”. This scenario highlights the central role of mitochondria as the 'powerhouse of immunity', as metabolism in immune cells shapes the body's response to infection [9]. This phenomenon has been primarily reported in lymphocytes, monocytes, and macrophages [25].

Lymphocytes react to cytokine stimuli initiated by monocytes, macrophages, dendritic cells, and neutrophils, which contribute to the innate immune response. Activated phagocytic cells, including monocytes and macrophages, release pro-inflammatory cytokines like interleukin-1 (IL-1) and IL-6 [26], primarily associated with energy deprivation [27]. Additionally, lymphocytes help to mitigate the potentially harmful consequences of the pro-inflammatory response, contributing to a favorable prognosis in sepsis [26]. Conversely, IL-10 expression plays a crucial role in modulating the immune system by inhibiting monocyte-macrophage activation and suppressing the production of tumor necrosis factor-alpha (TNF-α), IL-1, and interferon-gamma (IFN-γ) by lymphocytes [28]. In addition to cytokines signaling, the immune cells utilize intermediates of Krebs cycle, such as citrate and succinate, along with their derivatives like itaconate, to mediate anti- and pro-inflammatory gene expression [29]. Remarkably, metabolites derived from the Krebs cycle can influence the reprogramming of macrophages from the M1 phenotype (pro-inflammatory) to the M2 phenotype (anti-inflammatory). M1 macrophages exhibit impaired OXPHOS, while M2 macrophages possess an intact Krebs cycle, relying on OXPHOS as the primary source of ATP production [25].

These interactions between mitochondrial function and inflammatory response have been attributed to two major pathways. Firstly, the mammalian target of rapamycin (mTOR) pathway plays a key role in metabolic regulation by modulating glycolytic pathway. Moreover, the activation of mTOR and OXPHOS can induce metabolic reprogramming and the transition to oxidative glycolysis for energy production in CD4 + and CD8 + T cells [8]. The second is the nuclear factor kappa beta (NF-κβ) pathway, which is activated in response to stressors such as tissue damage, cytokine release, and pathogen-associated molecular patterns (PAMPs), increasing the expression of genes involved in the immune response. Upon activation of the NF-κB pathway and the subsequent induction of cytokine expression, macrophages differentiate into either M1 or M2 subtypes, depending on the cytokine milieu present at the site of infection [30].

**Fig. 1:**
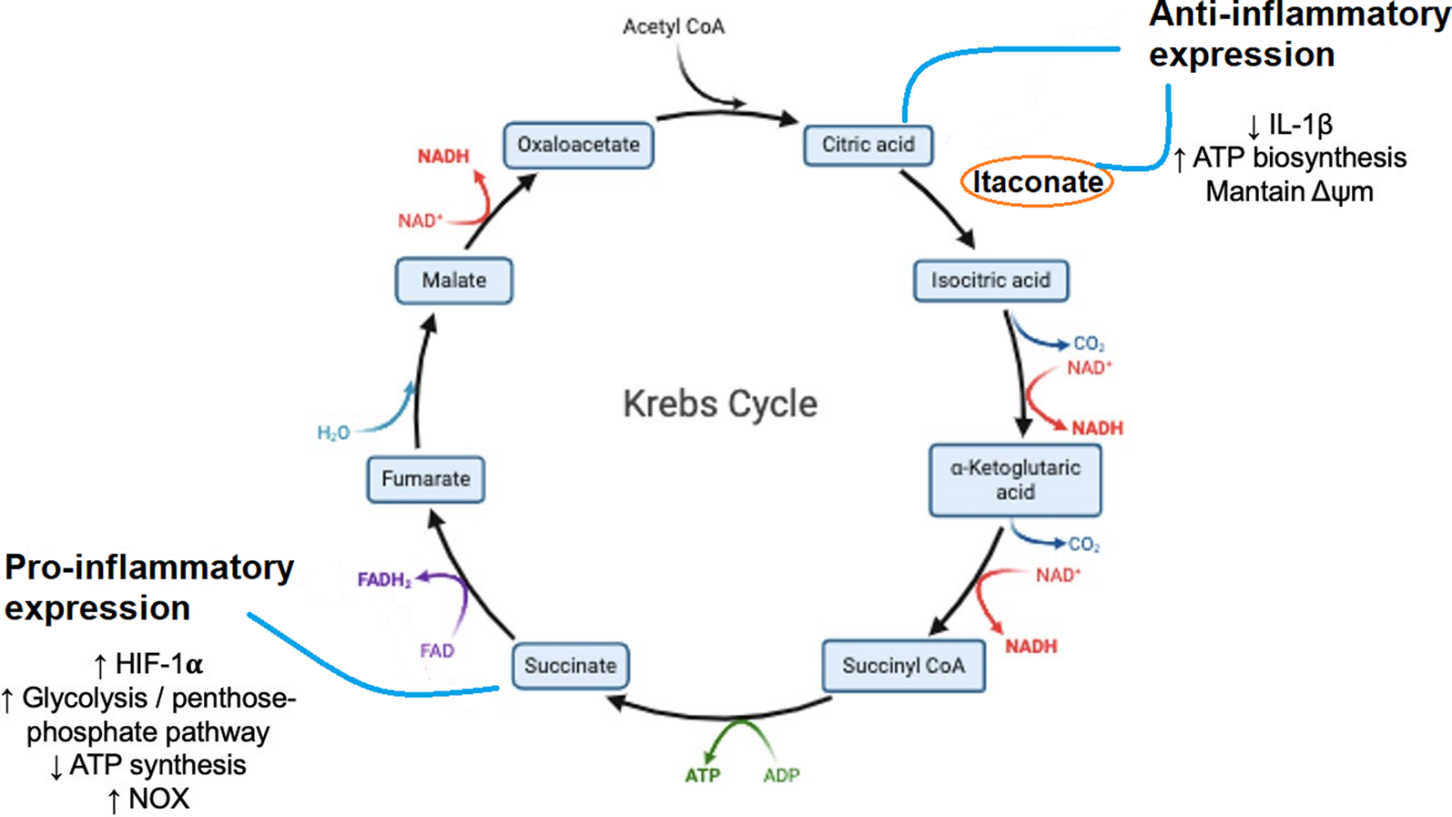
Inflammatory response mediated by mitochondrial metabolism in mononuclear cells. Krebs cycle intermediates, such as citrate and succinate, as well as itaconate, a metabolite derived from the decarboxylation of cis-aconitate, can activate pro- and anti-inflammatory responses. Δψm: protonmotive force; ATP: adenosine triphosphate; FAD: flavin adenine dinucleotide; FADH: flavin adenine dinucleotide (reduced); GDP: guanosine diphosphate; GTP: guanosine triphosphate; HIF-1α: hypoxia-inducible factor 1-alpha; IL: interleukin; NAD: nicotinamide adenine dinucleotide; NADH: nicotinamide adenine dinucleotide (reduced); NOX: NADPH oxidases

From a purely energetic perspective, pro-inflammatory polarization in innate immune cells promotes increased non-mitochondrial glucose metabolism and decreased OXPHOS, even when oxygen is abundant. This phenomenon is thoroughly reviewed in the literature [8]. While this shift in metabolism might initially seem maladaptive, current evidence suggests it may actually support innate immune functions rather than indicate mitochondrial dysfunction. For example, Britt et al. demonstrated that during the respiratory burst in neutrophils, the increased demand for nicotinamide adenine dinucleotide phosphate (NADPH) by NADPH oxidases (NOX) isoenzymes redirects glycolytic metabolites towards the pentose phosphate pathway (PPP), reducing substrate availability for mitochondria [31]. NOX activation is required for antigen-presentation, T-cell differentiation, B-cell proliferation, and cytokine production and release [32]. This suggests that the PPP rewiring described in neutrophils may also be relevant for other immune cells, and contribute to the reduced mitochondrial respiration in the context of sepsis. Furthermore, many studies report the diversion of the tricarboxylic acid cycle (TCA) cycle intermediates (citrate, succinate, fumarate, and itaconate derived from cis-aconitate) to the cytosol, where they act as immunomodulatory agents, independent of their energy-producing roles [33, 34]—Fig. 1. This suggests that the TCA cycle functions as an amphibolic modulator of immune cell responses, depleting metabolites for mitochondrial ATP production during intense pro-inflammatory activation and shifting metabolism towards OXPHOS during repair and recovery phases. New research is also uncovering additional components of the immunometabolic puzzle, highlighting unconventional pathways such as the aspartate-argininosuccinate shunt as significant mediators of mitochondrial and metabolic influence on immune responses [35]. This pathway, typically associated with the urea cycle, is activated in macrophages following lipopolysaccharide (LPS) stimulation. Further, such activation diverts mitochondrial oxaloacetate, limiting substrate availability for Complex I, and leads to the cytosolic accumulation of fumarate, and ultimately supports interferon-β production. While this mechanism has yet to be demonstrated in vivo, it could be a key element of the immunometabolic machinery contributing to decreased mitochondrial respiration and poor outcomes in septic patients.

## How to detect mitochondrial dysfunction in sepsis

**Fig. 2:**
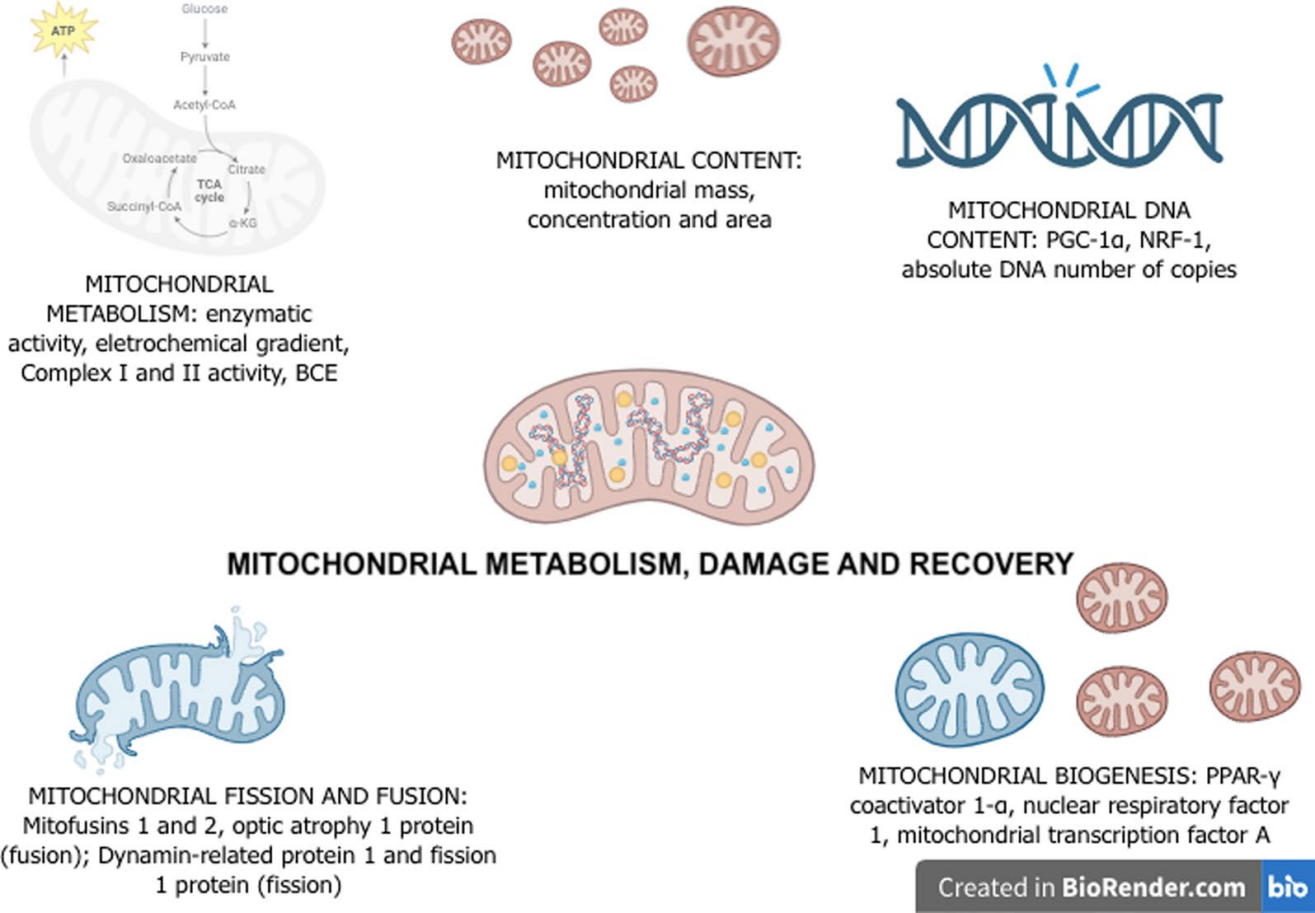
Mechanisms of mitochondrial damage, functional impairment and recovery. BCE: biochemical coupling efficiency; PGC-1α: peroxisome proliferator-activated receptor γ coactivator 1α; PPAR-γ: peroxisome proliferator-activated receptor gamma; NRF-1: nuclear respiratory factor 1

A detailed and accurate real-time analysis of mitochondrial malfunctioning in organs of living human patients in a hospital setting is impractical. Usually, mechanisms must be inferred from tissue specimens obtained from non-vital organs (e.g., blood and skeletal muscle), or from postmortem tissues examinations. Such specimens should carry out relevant biological signatures that mirror whether a mechanistic target is related to the severity of sepsis and to clinical outcomes. Whereas alterations in several dimensions of mitochondrial function in sepsis were already reported using preclinical tools, there is no recommended use for the hospital routine. Figure 2 presents an overview of the different ways to measure mitochondrial damage, activity and recovery in sepsis.

### Mitochondrial biogenesis

Mitochondrial biogenesis refers to the process by which cells generate new mitochondria, and depending on the demands also increases the number. This process requires the synthesis of mitochondrial proteins, which are encoded by nuclear DNA and subsequently imported and integrated into mitochondria [7]. Biogenesis further relies on the transcription of mtDNA, which encodes 13 genes that translate proteins to the respiratory complexes [37]. Biogenesis along with mitophagy serves to replace damaged/dysfunctional mitochondria, thus enhancing the ability to generate ATP in response to increased energy demands over time. In postmortem studies, septic patients exhibit mild-to-moderate mitochondrial swelling and increased markers of mitophagy in the kidney, with minimal cell death or indications of permanent damage, such as tissue fibrosis [36]. In addition, postmortem studies using liver and rectus abdominis muscle samples from critically ill patients have revealed an increase expression of transcriptional factors linked to mitochondrial biogenesis [37]. Similarly, it was found in biopsies of skeletal muscle of septic patients a partial activation of the mitochondrial biogenesis pathway involving NRF2α/GABP and its target genes, which does not parallel the benefit for mitochondrial function. However, research has also shown a decrease in mitochondrial content in the muscles of critically ill patients with sepsis-induced multiple organ failure [38]. Although these studies contribute to a better understanding of the mitochondrial signatures of the disease, they demanded muscle biopsy samples, which bring into light important practical limitations. However, these data suggest that biogenesis activation may play a role in the recovery phase of critical illness [39], albeit not yet correlating with improved mitochondrial function [17]. Hence, compromised mitochondrial biogenesis in critically ill patients, and activation of the biogenesis pathway may represent a key prognostic factor in critically ill patients associated with recovery of the initial injury [39].

Proteins involved in the regulation of mitochondrial morphology and maintenance of the fission–fusion balance are necessary for overall cellular health. Mitochondrial fission is necessary to preserve an ideal number of mitochondria during cellular growth and division processes, while fusion enables the unification of two mitochondria forming a more elongated one. In a homeostatic environment both fission and fusion processes result in healthy mitochondria [40]. It is tentative to assume that mitochondrial fusion and fission undergo alterations during the acute inflammatory response observed in sepsis. These changes can be assessed by measuring the levels of fusion proteins, such as mitofusins 1 and 2 and optic atrophy 1 protein, as well as fission proteins, including dynamin-related protein 1 and fission 1 protein [39]. Despite these proteins being promising biomarkers of mitochondrial dysfunction caused by sepsis, the extent of mitochondrial dynamics (fusion and fission) in sepsis patients remains largely speculative. In this context, new clinical studies may provide insights related to the association between dynamics and specific clinical outcomes in sepsis.

### Mitochondrial DNA

Mitochondria undergo various morphological changes during events such as fusion and fission, which help maintain a healthy mitochondrial population by facilitating the exchange of mtDNA, preserving mtDNA integrity, and regulating the size, quantity, distribution, thereby sustaining OXPHOS capacity [8]. These changes are also crucial for cell division and proliferation, as well as in the selective elimination of damaged or excess mitochondria through a process known as mitophagy [7]. Proteins involved in fusion and fission events, such as mitofusin-2 and dynamin-related protein-1, have been linked to changes in mitochondrial membrane potential and reduced oxygen consumption [41]. Fission and fusion processes become more prevalent under stressful conditions and play critical roles in eliminating damaged mitochondria and enhancing repair mechanisms. However, there is limited data regarding these mitochondrial dynamics in patients with sepsis, and the data may vary depending on the tissue type. From a theoretical standpoint, mitochondrial dynamics in patients with sepsis is a plasticity phenomenon, aimed at improving mitochondrial bioenergetic function, and decreasing oxidative stress in favor of cell survival [42].

However, mtDNA levels in the serum should be interpreted as a potential damage-associated molecular pattern that propagates an inflammatory response through interactions with the immune system [12, 43]. Therefore, mtDNA damage can lead to a pathological cycle, resulting in metabolic dysfunction, particularly in white blood cells [44]. The reduction in mtDNA content in the peripheral blood observed in the acute phase of sepsis could be only due to an increased concentration of neutrophils in the peripheral blood [45]. Also, it remains uncertain whether there is an interaction between mDNA concentration and changes in mitochondrial bioenergetics, particularly in immune cells. The assessment of serum levels of mtDNA is easy to perform and applicable, however, the role of these limitations and their prognostic impact must be better elucidated in new studies. Mitochondria possess their own DNA, and the depletion of mtDNA in an injury process may, hypothetically, cause defects in the ETC compromising OXPHOS [45, 46]. Another alternative is the organ-specific evaluation, through tissue biopsies, both in vivo and postmortem [47]. Although it is not a clinically applicable assessment, it allows for an organ-specific assessment regarding quantitative mtDNA polymerase chain reaction (qPCR) and mtDNA damage. Moreover, tissue utilization allows the evaluation of the interaction between mtRNA and mtDNA expression of mitochondrial biomarkers.

### Qualitative measurements of mitochondrial metabolism

In addition to alterations in mitochondrial mass, various hormones, enzymes, and regulatory pathways within cells are responsible for regulating the quality of mitochondrial function and promoting a shift towards glycolytic pathways, alternatively promoting the hierarchization of oxidative e non-oxidative metabolic pathways depending on the required pro- or anti-inflammatory response. Reactive derivatives of nitric oxide and superoxide anions, such as peroxynitrite, which cause oxidative stress, stimulate glycolysis by activating the rate-limiting step of the PPP, glucose-6-phosphate dehydrogenase. This pathway leads to the formation of NADPH in relation to NADH. NADH is a substrate for mitochondrial OXPHOS of high-energy phosphates, while NADPH is essential for the formation and repair of proteins, DNA, and lipids. By diverting glycolytic intermediates from the Krebs cycle and suppressing aerobic mitochondrial respiration, cells and tissues transition into a state of reduced oxygen consumption and ATP production. This phenomenon is commonly referred to as the “Warburg effect”, particularly in the context of cancer [48]: cells are less reliant on oxidative metabolism, thereby decreasing oxidative stress and promoting the formation of reducing equivalents (e.g., lactic acid and NADPH) that induce cell repair [49]. The Warburg effect and related mediators, such as hypoxia-inducible factor 1-alpha (HIF-1α), are induced in sepsis models, potentially providing cytoprotection and modulating inflammation in response to acute cell stress [50]. However, HIF-1α activation in immune cells can perpetuate the activation of the pro-inflammatory signaling through the stimulation of glycolytic pathway [51]. An estimation of PPP components, NADH:NAD^+^ ratio, and HIF-1α can be easily determined in immune cells through biochemistry assays, reactive polymerase chain reaction, and Western blotting.

Proton pumps of the ETC, in conjunction with F1Fo-ATP synthase, establish a proton gradient across the inner membrane, generating both an electrochemical potential [proton motive force (pmf), in mV] and a flux of protons (proton current in nmol of protons/min). Mitochondrial membrane potential is a critical component of healthy mitochondrial metabolism and contributes to determining the pmf [10]. Thus, measuring the pmf serves as a surrogate marker of mitochondrial functionality. This methodology, however, is currently feasible only in isolated mitochondria and cells, is relatively time-efficient, and, although valuable for understanding disease mechanisms, has yet to be integrated into clinical practice.

Reductions in both the expression and activity of Complexes I, II, III, and IV, as well as of cytochrome c (Cyt c) have been reported in critically ill patients [15, 39]. However, it is still doubtful whether these alterations are determinants of the patient's prognosis, or if they are just epiphenomena in the acute context of critical illness. However, they are useful and commonly used to assess mitochondrial activity [52–55], especially when normalized by enzymatic activity, protein, or DNA concentration. This type of measurement can be done on circulating cells (mononuclear cells, platelets) or on biopsy-derived cells (muscle cells, for example). Relative content of mitochondrial proteins are determined by Western blotting, using primary antibodies for mitochondrial complexes subunits. Also, Cyt c content can also be quantified in immunoassay of isolated cells.

### Respirometry

Quantifying the efficiency of mitochondrial ATP production is a logical method for evaluating their functional integrity. Measuring mitochondrial respiration is a cost-effective and time-efficient approach compared to conventional methods of assessing mitochondrial function through biopsies, making it widely accessible. High-resolution measurements of mitochondrial respiration can be obtained using advanced instruments equipped with highly sensitive microcathode oxygen electrodes, which can be utilized in acute care settings. The measurement of mitochondrial respiration can be performed using the substrate–uncoupler–inhibitor titration (SUIT) protocol, which is a commonly used method for quantifying this process. This protocol entails the titration of various combinations of substrates, uncouplers, and inhibitors in order to evaluate mitochondrial respiratory function [11]. The SUIT protocol permits the investigation of intricate interactions between coupling and substrate control in a single assay, thereby measuring multiple aspects of mitochondrial physiology [56].

**Fig. 3:**
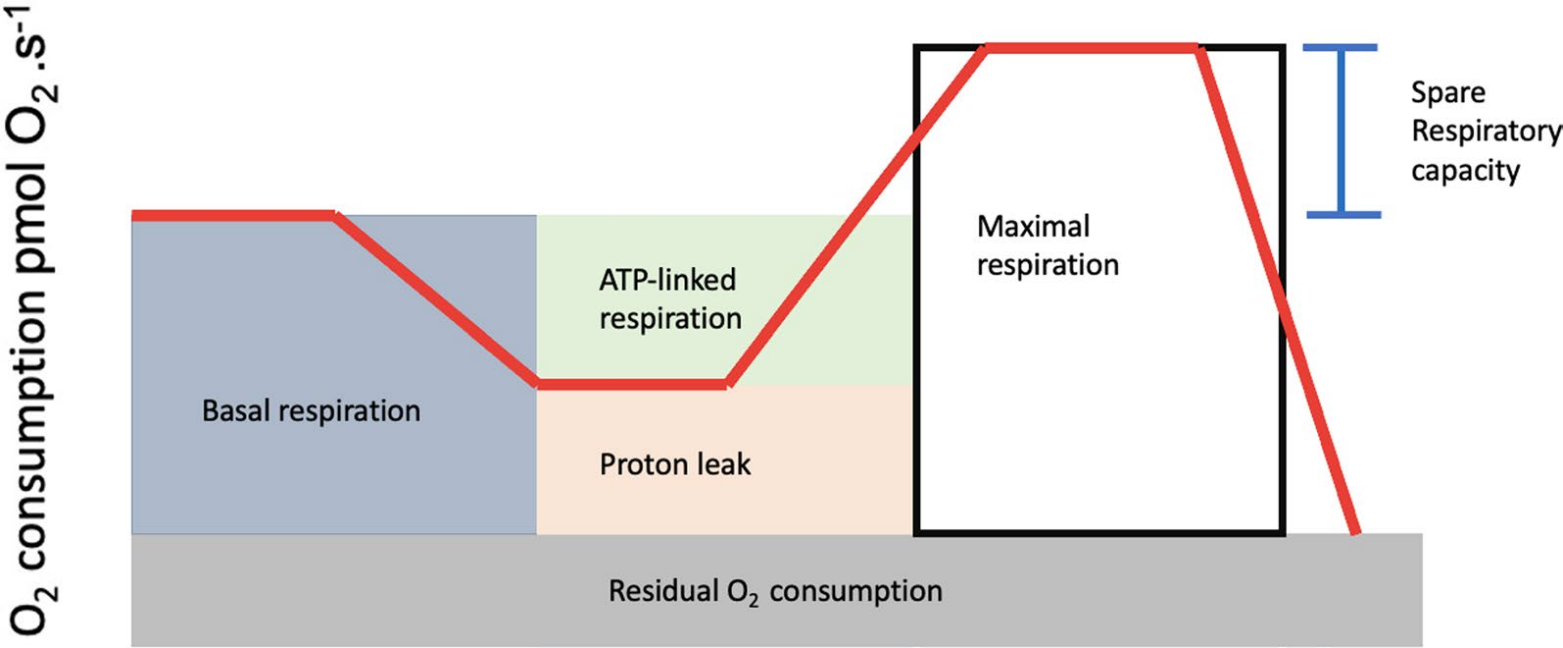
Example of a respirometry assay. ATP: adenosine triphosphate

Respirometry allows for real-time measurement of mitochondrial respiration, providing crucial parameters through the application of established inhibitors and uncouplers, which serve as sensitive indicators of the response to mitochondrial stress. Figure 3 depicts the mitochondrial respiration trace derived from the SUIT protocol, which was employed in the following procedures [8, 56]: (1) routine respiration: routine respiration, also known as basal respiration, measures the oxygen consumption resulting from ATP production and proton leak. This represents energy demand under steady-state conditions. Changes in routine respiration in patients with disease compared to controls may indicate altered mitochondrial function and should be interpreted in the context of the following mitochondrial parameters: (2) proton leak: after measuring routine respiration, cells are exposed to oligomycin, an inhibitor of Complex V. The remaining mitochondrial respiration after the addition of oligomycin was attributable to proton leak. Although some proton leaks are expected under physiological conditions, significant proton leak may indicate damage to the mitochondrial membrane and/or complex damage. The use of oligomycin also allows for the estimation of oxygen consumption secondary to ATP production, often referred to as ATP-linked respiration. (3) Maximal respiration: the addition of a mitochondrial uncoupler, such as dinitrophenol or carbonyl cyanide-4-(trifluoromethoxy) phenylhydrazone, stimulates maximal respiration by mimicking the physiological energy demand, leading to an increase in oxygen consumption. The difference between maximal respiration and routine respiration represents the spare respiratory capacity (SRC) of the cell. SRC indicates the ability of a cell to respond to energetic stress and is a measure of a cell’s fitness. A decrease in SRC may limit the cell's ability to handle stressors, resulting in mitochondrial dysfunction. (4) Residual oxygen consumption; the addition of mitochondrial inhibitors, such as the combination of rotenone (Complex I) and antimycin (Complex III), completely inhibits the electron transport system. The remaining oxygen is consumed by non-mitochondrial respiration in the form of oxidases and other cellular enzymes that utilize oxygen. Residual oxygen consumption may increase in the presence of a stress response.

Under normal conditions with excess ADP and oxygen, mitochondrial respiration, known as state 3 respiration, occurs rapidly. Conversely, when ADP was fully consumed, state 4 respiration occurred, which was significantly slower. State 4 respiration can be induced by “uncoupling” oxygen consumption from OXPHOS, leading to proton leakage back into the mitochondrial matrix without the production of cellular energy. One of the most promising indicators of mitochondrial function is biochemical coupling efficiency (BCE), which is calculated as the quotient between OXPHOS and proton leak. BCE reflects the true effectiveness of mitochondria in utilizing oxygen for ATP production [8, 75]. This is a useful way to gain more insight into the site of the dysfunction, namely, respiratory control decreases because of dysfunction in localized sites of substrate oxidation, ATP synthesis, proton conductance, or F1Fo-ATP synthase [10].

Although several centers may not have access to equipment for real-time measurement of mitochondrial respiratory rates, this limitation can be easily overcome by utilizing simpler biochemical colorimetric methods that maintain a reasonable level of sensitivity and offer short assay times, which can be easily integrated into the hospital laboratory routine. For example, measurements of succinate dehydrogenase enzyme activity for Complex II (succinate: DCIP-oxidoreductase), Complex IV, and Complex V are routinely performed in research laboratories [76, 77]. However, these aforementioned colorimetric methods only allow for the evaluation of metabolic activity in a single complex at a time, while mitochondrial respirometry assays enable the simultaneous assessment of the metabolic activity of multiple complexes. This approach has the potential for clinical and large-scale applicability, but further studies are necessary for its validation.

## Mitochondrial metabolism measurements in different organs and systems

We searched PubMed from database inception to June, 2024, using the search terms: ‘diaphragmatic dysfunction’ AND (‘mitochondria’ OR ‘mitochondrial dysfunction’) AND ‘sepsis’; ‘septic cardiomyopathy’ AND (‘mitochondria’ OR ‘mitochondrial dysfunction’); (‘septic acute kidney injury’ OR ‘septic AKI’) AND (‘mitochondria’ OR ‘mitochondrial dysfunction’). We also searched for the terms (‘mitochondria’ OR ‘mitochondrial dysfunction’ OR ‘mitochondrial impairment’) AND (‘sepsis’ OR ‘septic shock) for clinical studies (cohort studies or clinical trials) exploring sepsis prognosis and mitochondrial metabolism. The evaluation of the prognosis of mitochondrial dysfunction in sepsis was carried out focusing on the association between mitochondrial dysfunction with mortality and recovery from sepsis in critically ill patients. Returned lists of articles were then screened manually by reading abstracts. Comprehensive reviews that have been published within the past 5 years were also read in full, and their reference lists were reviewed. Remaining manuscripts were read in full and their references reviewed when appropriate.

Although it is logical that dysfunction in mitochondrial metabolism can lead to a certain degree of organ failure, measuring it is often not feasible due to ethical questions, costs, and the difficulty of obtaining adequate mitochondrial samples from vital organs in critically ill patients [74]. Therefore, it is feasible to assess mitochondrial dysfunction in cells that are easy to collect, especially those that may reflect the “systemic” effects of sepsis. Peripheral blood cells have been used in translational research to assess bioenergetic functions. Peripheral blood mononuclear cells (PBMCs) are mitochondria-rich with high rates of respiration [18]; thus, they are excellent candidates in circulating blood to provide a reliable estimation of global oxidative metabolism, particularly metabolism linked to the immune response. Lymphocytes comprise the majority of PBMCs and are traditionally used to measure defects in mitochondrial OXPHOS [35]. The exhaustion of lymphocytes, especially T cells, leads to an increased risk of secondary infections, which is correlated with mortality [78]. Circulating immune cells play an essential role in the pathophysiology of sepsis, as their activation may remotely induce inflammation in noninfected organs [79]. The measurement of mitochondrial respiration in PBMCs of septic patients has the potential to identify those at risk of negative outcomes, monitor the clinical course and response to treatment, and serve as a useful marker for acute care settings [11]. Moreover, this approach allows researchers to unravel the mechanisms associated with impaired metabolism that follow this syndrome and challenge whether this could be improved by therapeutic intervention.

**Fig. 4:**
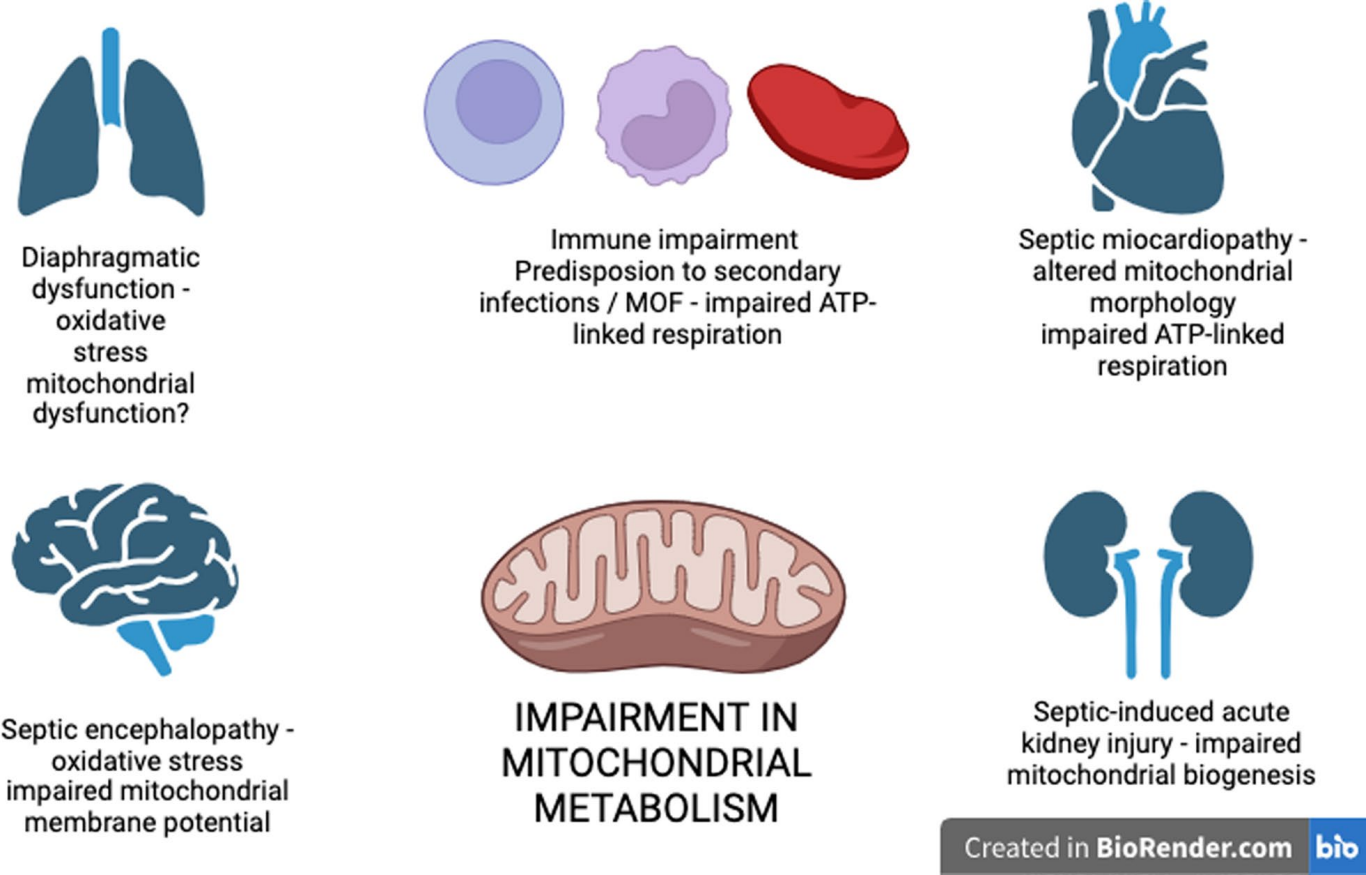
Mitochondrial dysfunction and its association with different organ failures in sepsis

As explained previously, evaluating mitochondrial and cellular function in specific organs is often a limitation from a clinical perspective. Studies evaluating the mitochondrial metabolism in organs are most derived from animal models of sepsis. In this context, mitochondrial dysfunction has been described in different disease scenarios in intensive care and is associated with different organ failures (Fig. 4).

Diaphragm atrophy and diaphragmatic dysfunction prolong mechanical ventilator dependency, increasing morbidity and the duration of hospital stay [57]. In animal and cultured muscle cell studies, diaphragm dysfunction is strongly associated with oxidative stress, which, when transiently increased, may represent a potentially protective process, whereas uncontrolled persistence or high concentrations of ROS may result in diaphragm atrophy and dysfunction [18]. However, the impact on mitochondrial respiration remains uncertain. In a small study exploring diaphragm muscle fibers isolated from biopsies of mechanically ventilated critically ill patients, diaphragm fiber atrophy and weakness were not associated with impaired mitochondrial respiration [57]. In a study of sepsis-induced mice, there was downregulation of respiratory chain complex mRNA expression, decreased Complex III and IV protein levels, and reduced oxygen consumption associated with ADP phosphorylation [58]. Mitochondria are abundant in the heart and are responsible for significant ATP production. Since abundant ATP maintains contraction and diastolic function of the heart, mitochondrial dysfunction is linked to septic myocardiopathy due to energy deficits [19]. Morphological changes in myocardial mitochondria can be observed early during sepsis, and within 24 h post-LPS infusion, there is also an absolute reduction in the number of mitochondria [59]. In LPS-induced rat models, increased ROS levels cause mitochondrial respiratory dysfunction, which leads to septic cardiac disease and performance [60]. In septic myocardiopathy, an impairment in the mitochondrial respiration rate is also observed, with a decreased state 3 respiration rate (suggesting a suppressed ETC) and an elevated state 4 respiration rate, suggesting mitochondrial damage in association with suppressed respiratory chain complex enzymes [59]. Through bioinformatics analysis approaching to identify potential interactions in septic myocardiopathy, it was found specific mitochondrial expressed genes associated with septic cardiomyopathy and inflammatory pathways mediated by monocyte and B cells, thereby confirming pathophysiological interactions [61].

Mitochondria are the main consumers of oxygen in kidneys. Dysfunction in energy metabolism is a critical contributor to the pathogenesis of acute kidney injury (AKI) [47], and mitochondria could serve as a potential therapeutic target. Several mitochondrial mechanisms are involved in septic AKI. Firstly, the energetic metabolism is impaired, with a reduction in OXPHOS rate and an increase in ROS and in glycolysis. Despite the fact that glycolysis provides less efficient energy generation, it can provide sufficient energy for cell survival as well as for maintenance of essential structural components. ROS per se is toxic to the endothelium, and defense mechanisms are critical to maintain organ perfusion and function [62]. Later activation of adenosine monophosphate-activated protein kinase (AMPK), a master sensor of energy catabolic status, may allow for cell survival and mitochondrial biogenesis. Moreover, synthesis of new mitochondrial mass may be an important mechanism for recovery from septic AKI [63]. Mitochondrial biogenesis can increase ATP production in response to increasing energy demand by the generation of new and functional mitochondria, and PGC-1 $\propto$ is a positive mitochondrial biogenesis regulator [62]. During sepsis, na increase in TNF-$\propto$ levels can reduce PGC-1 $\propto$ expression in tubular cells and suppress kidney recovery. Changes in mitochondrial morphology, with fragmentation of organelles, are also observed in tubular cells along the sepsis course.

The brain is one of the most active organs in the body and exhibits a high energetic metabolism. The predominant source of energy consumed by the brain relies on the oxidation of glucose [64]. The sepsis-induced systemic inflammation can affect blood–brain barrier (BBB) function. The resulting BBB permeability allows peripheral immune cells from the bloodstream to enter the brain, which then release a range of inflammatory mediators and activate glial cells. The activated microglia and astrocytes release ROS, cytokines, chemokines, and neurochemicals, resulting in exacerbate inflammatory milieu in the brain [65]. ROS-induced oxidative stress causes mitochondrial dysfunction in neurons by altering the membrane potential of mitochondria and nitration of mitochondrial proteins [66]. In animal models, the occurrence of septic encephalopathy is closely related to mitochondrial dysfunction due to dysfunctions in ETC in mitochondria [67], which mediate intrinsic apoptosis in neural cells induced by sepsis due to the release of Cytochrome c [64].

## Mitochondria as a playmaker in septic multi-organ failure

Various systemic inflammatory processes can exert different effects on mitochondria. In the early stages of sepsis, reduced perfusion resulting from intrinsic and extrinsic fluid losses, decreased intake, myocardial depression, microcirculatory redistribution of blood flow, and loss of vascular tone, can lead to tissue hypoxia. This condition, characterized by insufficient oxygen availability at the mitochondrial level, impedes the ability of mitochondria to carry out OXPHOS, leading to a deficit in ATP production [12]. Although Complex IV exhibits distinctive enzyme properties that facilitate its functionality under hypoxic conditions, severely diminished oxygen concentrations may compromise ATP generation and activate cell death pathways, thereby adversely impacting cellular homeostasis [68, 69]. Also, this condition may lead ATP synthase to work in a reversal manner, thus consuming ATP rather than synthesizing it. Hormonal alterations in sepsis also affect mitochondrial function and efficiency. For example, thyroid hormones are believed to work predominantly through the modulation of mitochondrial activity [70]. Thirdly, genes that transcribe mitochondrial proteins are downregulated early in the inflammatory response. This was first recognized in human volunteers receiving endotoxins [71] and subsequently described in critically ill patients [52].

### Clinical studies exploring mitochondrial dysfunction in sepsis

Different studies, in different contexts, have evaluated mitochondrial activity in septic compared to non-septic or control patients. Muscular cells are prone to impaired mitochondrial metabolism during critical illness [72] and are an important research field. Carré et al. [52] used muscle tissue biopsies from critically ill patients, comparing them to those of controls subjected to hip surgery. They found a decrease in mitochondrial density in critically ill patients, without a decrease in Complex I and Complex IV activity. In a study evaluating patients with ICU-acquired weakness, comparing ATP synthesis in this population with metabolically healthy controls, ICU patients had an approximately 50% reduction in the ability of skeletal muscle to synthesize ATP in mitochondria, with a depletion of Complex III and IV mass [73]. A similar loss of mitochondrial activity was detected in a previous study in a population with sepsis and multi-organ failure [74]. Complex I and Complex IV activity was reduced in the intercostal and leg muscles, respectively, compared to controls.

Belikova demonstrated a higher baseline PBMC oxygen consumption and attenuated response to ADP stimulation in patients with sepsis than in healthy volunteers [75]. In blood mononuclear cells, Kraft et al. [17] demonstrated an early sepsis-mediated disruption of mitochondrial quality control in septic patients, with a later activation of mitochondrial biogenesis in this population. These patients also showed increased mitochondrial damage (measured by mtDNA levels) during the early phase of sepsis management. In a cohort of septic and non-septic ICU patients with measurement of mitochondrial function in isolated lymphocytes, critically ill patients had increased mitochondrial oxygen consumption but no significant difference in mitochondrial membrane potential [76]. Jang et al. [16] also found a lower routine, uncoupled Complex I, and maximal respiration in septic patients, when compared to controls in the early sepsis management. A respirometric study in PBMC developed by Japiassú et al. [77] reported reduced F1Fo-ATP synthase activity, thereby reducing ATP production. This may contribute to the energetic failure reported in these cells during the course of septic insult. In addition, septic shock PBMC have reduced O_2_ consumption, ADP-induced state 3 respiration, and respiratory control ratio compared to control PBMC. Inhibition of Complexes I, III, and IV in PBMCs from septic patients compared with controls was also detected in another study [78]. In a pediatric population, Weiss et al. [79] detected a decrease in spare respiratory capacity on days 1–2 of sepsis compared with controls. Spare respiratory capacity normalized on days 5–7. Patients with sepsis also had a higher ratio of leak to maximal respiration than controls, with normalization in the later phase of sepsis. Patients with sepsis did not show differences in basal or ATP-linked oxygen consumption or membrane potential.

In patients admitted to the emergency department with and without sepsis, Puskarich [80] did not find differences in plasma levels of cytochrome B, NADH, and Cox-III mtDNA between groups. Pyle et al. [45], in a protocol that evaluated mononuclear cell mtDNA content, found that these levels were lower in patients with sepsis, with depletion of monocyte and lymphocyte mtDNA. Platelet studies have also evaluated mitochondrial metabolism in patients with sepsis. Sjövall et al. [55] found an increase in state 3 and a decrease in RCR in patients with sepsis compared to controls during sequential evaluations in the first week of sepsis diagnosis. Additionally, patients with sepsis had increased rates of Complex I and Complex II respiration compared to controls. However, the mtDNA concentration did not differ between the platelets of patients with sepsis and controls.

### Are mitochondrial metabolism impairments associated with mortality?

A criticism can be made of the differences in mitochondrial measurements between survivors and non-survivors observed in studies. Whether they are pathological or just another measure of disease severity requires further investigation [80]. In human subjects, a significant constraint of this research approach is the uncertainty surrounding whether mitochondria sourced from PBMCs, platelets, or muscle cells can accurately serve as proxies for the mitochondria present in essential organs like the liver, kidneys, and heart [80]. The establishment of a workable, all-encompassing strategy to investigate the complete energy production pathway in human beings would signify a more significant achievement, as it would facilitate a deeper comprehension of the origin of lactate in distinct patients and across time, thereby enabling more targeted clinical trials for novel treatments for bioenergetic dysfunction.

Defects in leukocyte energy metabolism [81], particularly in T lymphocyte cells [82], are intrinsically associated with the state of immunoparalysis in sepsis. Metabolic events in the mitochondria of macrophages, dendritic cells, and T-lymphocytes have profound effects on immunity. When exposed to infectious injury, OXPHOS levels decrease, with a concomitant increase in glycolysis. An outcome of the reduced ATP production via OXPHOS is the redirection of mitochondria towards generating mitochondrial ROS and anti-inflammatory signaling, which function as signaling molecules essential for eliciting an appropriate immune response [44]. Therefore, mitochondrial respiration is essential for the functioning of these cells.

Despite the fact that the current knowledge suggests a potential role of mitochondrial metabolism impairment in septic patients (when compared with controls) with a potential impact on prognosis, the literature is heterogeneous with regard to the findings of mitochondrial dysfunction (Table 1). It is still necessary to define the most practical way of measuring, with the greatest clinical applicability, the greatest prognostic impact, and, above all, the most accurate in predicting the clinical course of the disease.

**Table 1:** Studies that explored association of mitochondrial dysfunction and prognosis in sepsis

Study	N of critically ill septic patients	Mitochondrial measurement	Main findings (survivors vs non-survivors)
Rogers et al. [86]	42	Metabolomic of protein translation (N-formyl-l-methionine)	There was increased levels of N-formyl-l-methionine in septic shock non-survivors when compared with survivors measured within 24 h of ICU admission
Brealey et al. [54]	28	ATP concentration, Complex I, II, and IV activities in biopsied muscle cells	Sepsis survivors had an increased level of ATP and Complex I and IV activity
Carré et al. [52]	16	Mitochondrial morphology (surface density and volume), RT-PCR of mitochondrial biogenesis factors and concentration of Complex I and Complex IV mitochondrial proteins and OXPHOS transcripts in muscle biopsy specimens	Nonsurvivors had an increased decline in mitochondrial surface density, with a similar mitochondrial volume in these groups; OXPHOS transcripts were more abundant in survivors; increased ATP content in survivors; no difference between groups in Complex I and Complex IV activity
Kraft et al. [17]	37	qRT-PCR for genes that regulate mitochondrial biogenesis in PBMCs	Increased genetic activation of mitochondrial biogenesis in day 3 compared with day 1; decrease in mtDNA in septic patients compared with controls, with a recovery on day 5; early activation of mitochondrial biogenesis by day 1 associated with ICU discharge; increased mRNA levels in survivors
Japiassú et al. [77]	20	Respirometry of PBMC evaluating state 3, 4 and respiratory control ratio	No difference in ADP-stimulated respiration in non-survivors, when compared with survivors
Nedel et al. [15]	90	Respirometry of permeabilized lymphocytes	Improvement in Complex I, Complex II, basal and in BCE in day 3, compared with day 1, were associated with lower mortality. In multivariate analysis, BCE improvement was associated with lower 6-mo mortality
Puskarich et al. [80]	28	Respirometry of platelets	Routine and state 3 respiration were significantly higher in non-survivors compared to survivors; state 4 respiration had a non-significant increase in non-survivors
Pyle et al. [45]	Not reported (147 patients, including septic)	mtDNA content from mononuclear cells	No relationship between mtDNA content and survival outcome at 180 d
Sjövall et al. [55]	18	Respirometry of isolated platelets evaluating Complex I, state 3, 4 and respiratory control ratio	Nonsurvivors had an increased Complex I, Complex II, state 3 respiration and an increased respiratory control ratio at day 6–7 of sepsis when compared with survivors
Sjövall et al. [87]	20	Respirometry of permeabilized peripheral blood immune cells	Survivors and non-survivors at 90 d after sepsis did not have difference in Complex I plus Complex II respiration normalized to citrate synthase, mtDNA, and cytochrome c
Li et al. [88]	20	Analysis of mitochondrial-associated genes	Lower expression levels of mitochondrial genes COX7B and NDUFA7 were associated with a lower 28-day mortality in sepsis patients
van der Slikke et al. [89]	91	DNA and RNA oxidated in urine mass-spectrometry and plasma levels of mtDNA	RNA oxidation is an independent predictor of long-term all-cause mortality
Robles et al. [90]	24	Respirometry or PBMCs	Survivors had higher routine respiration and maximal respiration compared with non-survivors
Huang et al. [91]	133	Fis1, parkin, mitofusin2, PGC-1α	Fis1/parkin ratio, Fis1/mitofusin2 ratio, Fis1/PGC-1α ratio and Fis1 ratio were lower in 28-day survivors when compared with non-survivors. Fis1/parkin ratios was an independent risk factor for 28-day mortality
Busani et al. [92]	19	Plasma circulating mtDNA levels	Patients who survived had lower levels of mtDNA when compared with non-survivors
Hernández-Beeftink et al. [93]	687	Plasma circulating mtDNA levels	mtDNA levels were associated with 28-day mortality in septic patients who developed ARDS

ADP: adenosine diphosphate; ARDS, acute distress respiratory syndrome; ATP: adenosine triphosphate; BCE: biochemical coupling efficiency; Fis1: fission1 protein; ICU: intensive care unit; qRT-PCR: quantitative reverse transcriptase-polymerase chain reaction; mtDNA: mitochondrion desoxyribonucleic acid; OXPHOS: oxidative phosphorylation; PBMC: peripheral blood mononuclear cells; PGC-1α, peroxisome proliferator-activated receptor γ coactivator 1α; RNA, ribonucleic acid

### Are mitochondrial metabolisms associated with recovery of sepsis?

Therefore, mitochondrial biogenesis is critical for recovery, and the recovery from organ dysfunction is preceded by an increased mitochondrial biogenesis [70]. In a study of patients with multi-organ failure in intensive care, patients that survived had higher levels of PGC-1α and better-preserved levels of ETC Complex protein, along with a more robust antioxidant response in the early stages of their disease progression [52]. The ability to clear damaged mitochondria is another important phenomenon [83]. Mitophagy (autophagic degradation) and mitoptosis (programmed destruction) are the processes by which cells remove damage with impaired mitochondria [12]. The efficiency of these processes may be an important contributing factor to the pathogenesis of various states of the disease. The process of mitophagy entails the targeted sequestration and subsequent degradation of damaged mitochondria, which occurs prior to their ability to activate cell death pathways and potentially jeopardize the viability of the entire cell. Thus, mitophagy operates as an initial protective response. Conversely, heightened levels of oxidative stress and apoptotic proteases can impede the function of mitophagy and stimulate additional inflammatory responses [84].

Along with a sepsis episode, there is an immunosuppressive state that can persist for weeks or even months after critical illness, even after sepsis recovery. This state of immunosuppression can contribute to poor long-term outcomes [85]. Mitochondrial dysregulation is tightly associated with these immune cells dysfunction [8]. Strategies to recover the balance between oxidative and non-oxidative metabolism in immune cells may favor the restoration to a normal immune response. Functional recovery of mitochondria, expressed in an increase in ATP-linked oxygen consumption in peripheral blood mononuclear cells correlates with improved outcomes in septic patients [15]. This encompasses an important characteristic for being explored as a prognostic and therapeutic monitoring biomarker.

## Conclusion

Although evidence underpinning mitochondrial dysfunction is consistent, the exact nature is conflicting and relates to heterogeneity in terms of timing, immune cell type, cell or animal model or patient and differing research methodologies. Acute inflammatory conditions, such as sepsis, can lead to alterations in mitochondrial components, including protein content, mtDNA concentration, oxidative complexes activity, and F1Fo-ATP synthase, and immunometabolic signaling molecules. These alterations can have significant clinical implications. Given these findings, it is important to further explore the role of mitochondria as a potential biomarker in prognosis, sepsis rehabilitation, and its association with different spectrums of organ failure.

## Data Availability

Not applicable.
